# Modern contraceptive utilization and its associated factors in East Africa: Findings from multi-country demographic and health surveys

**DOI:** 10.1371/journal.pone.0297018

**Published:** 2024-01-19

**Authors:** Henok Demeke, Nanati Legese, Shambel Nigussie

**Affiliations:** College of Health and Medical Science, School of Pharmacy, Haramaya University, Harar, Ethiopia; UiA: Universitetet i Agder, NORWAY

## Abstract

**Background:**

The use of modern contraceptives has been low in most Sub-Saharan African countries despite high population growth and a sluggish economy. This study aimed to identify the prevalence and determinants of modern contraceptive use among married reproductive-age women in East Africa.

**Methods:**

For this study, the Demographic and health survey (DHS) data from nine countries in East Africa were analyzed, yielding a weighted sample of 32,925 married women. A multilevel mixed-effect logistic regression model was used to identify characteristics associated with the utilization of modern contraceptives at a p-value less than 0.05. For model comparison, we used the Akaike and Bayesian Information Criteria (AIC and BIC). For assessing variation (random effects), we used community-level variance with standard deviation and intra-cluster correlation coefficient (ICC).

**Results:**

The overall prevalence of modern contraceptive use was 45.68%, 95% CI (45.15, 46.21). Women’s age, maternal education level, husband education level, media exposure, wealth status, occupation, religion, the total number of children ever born, distance to health facilities, history of termination of pregnancy, couple’s desire for children, women’s participation in decision making, living country and place of residence were significantly associated with modern contraceptive use in Eastern Africa.

**Conclusions:**

Conferring to this study, utilization of modern contraceptives is low in East Africa. Interventions to improve the use of modern contraceptives should encompass disseminating awareness through mass media, enrolment of males in family planning, giving maternal education, building health facilities in remote areas, and encouraging family planning programs in rural areas.

## Introduction

Family planning is a service that allows people to decide whether to have children by educating, supporting, and proposing birth control methods [1]. Family planning has been related to numerous welfares by reducing pregnancy-related risks [2, 3]. In developing countries, contraceptive usage disallowed 218 million unintended pregnancies, 55 million unintended births, and 138 million abortions in 2012, among which 40 million were risky, 25 million were miscarriages, and 118,000 maternal deaths [4].

The forms of contraceptive methods for family planning are divided into modern contraceptive methods and traditional methods [5]. Modern contraceptive methods comprise; short-acting contraceptives (injectable, pills, male condoms, female condoms, emergency contraceptives), long-acting reversible contraceptives (hormonal implants and intrauterine devices), and permanent contraceptives (female sterilization and male sterilization) [6–10].

Notwithstanding the worth of refining maternal and child well-being, an enormous number of women do not utilize contraceptives, with notable disparities covering wide geographic zones [11, 12].

In 2017, around 63% of married women of reproductive age or their partners used either modern or traditional means of contraception [13–15]. In the past 20 years, the use of modern contraception has enlarged to some extent, from 54.4% to 57.4% in the globe, according to the United Nations Population Fund [12, 16].

In 2017, the contraceptive prevalence was 58% in Oceania, whereas it was 75% in the Caribbean, Northern, and Latin America. Nevertheless, parallel to other regions, there is a low prevalence of contraceptive consumption in Africa relative to the use of modern contraceptives among married women who have the aim to postpone or avoid pregnancy [13].

The utilization of modern contraceptives is affected by various factors. Several studies on factors associated with the use of contraceptives have been conducted in some developing countries [17]. Various studies revealed the influence of individual factors and community factors on the use of contraceptives. Age of women [17, 18], number of alive children [14], place of residence [19], exposure to media [20], place of residence, and region [21] are among crucial determinants associated with modern contraceptive usage.

This study aimed to determine the prevalence of modern contraceptive use and to identify what factors are related to the usage of modern contraceptives among reproductive-age women in Eastern Africa. Unlike the previous study, we used the couples recode file (married women as the study population). The findings of this study are expected to assist stakeholders and policymakers in minimizing maternal mortality and morbidity by identifying significant points of challenges that provide keys to accelerating the use of modern contraceptives.

## Methods

### Study setting and data source

According to the United Nations (UN) Statistics Division, the African continent has five regions. Among these, East Africa includes 19 countries (Burundi, Comoros, Djibouti, Ethiopia, Eritrea, Kenya, Madagascar, Malawi, Mauritius, Mozambique, Reunion, Rwanda, Seychelles, Somalia, Somaliland, Tanzania, Uganda, Zambia, and Zimbabwe) and it is one of the largest regions.

Among these, DHS was not conducted in 6 countries (Djibouti, Somalia, Somaliland, Seychelles and Mauritius, Reunion), while it was conducted before 2015 in 4 countries (Eritrea, Comoros, Kenya, and Mozambique).

In this study, we included the recent DHSs from 9 East African countries (Burundi, Ethiopia, Madagascar, Malawi, Rwanda, Tanzania, Uganda, Zambia, and Zimbabwe). The DHS for these countries was conducted from 2015 to 2021.

We accessed the data from the DHS official database (www.measuredhs.com) after we were authorized for use (S1 File). The data was accessed on 05/20/2023. To extract the dependent and independent variables, we used the Couples Recode (CR) data set. The DHS program adopts standardized methods involving uniform questionnaires, manuals, and field procedures to collect data comparable across countries.

DHS is a nationally representative household survey that offers data from a wide variety of population, health, and nutrition tracking and effect assessment measures with face-to-face interviews of women aged 15 to 49. The sampling method for the DHS was a stratified multi-stage random sampling. The study population was women aged 15 to 49 years. Detailed survey techniques and sampling methods are recorded elsewhere [22].

### Variables

#### Outcome variable

The use of modern contraception was analyzed for married women aged 15–49 years who reported that they were currently on modern contraceptive use. The outcome variable in this study is modern contraceptive use. Modern contraceptive use refers to whether a woman was using methods such as oral pill, intrauterine device, condom, female or male sterilization, implant, injectable and other modern methods at the time of the survey. Analyzing modern contraceptive use is critical since it helps stakeholders understand the magnitude and factors that significantly affect use; hence, it will be easier to intervene on the factors. We created a binary variable in which the use of modern contraceptives was assigned “yes” (coded as 1) and not using modern contraceptives was assigned “No”(coded as 0).

#### Independent variables

Based on different literature, two types of independent variables were considered (individual-level and community-level). The community-level variables include country and residence. The individual level variables include maternal age, wealth status, religion, occupation, woman’s and her husband’s education level, sex of household head, way of last delivery, history of termination of pregnancy, family size, parity, gravidity, ideal number of children, desire for more children, mass media exposure, women’s empowerment (participation in decision making) and distance to health facility.

To measure exposure to mass media, watching television (TV), listening to radio, and reading newspapers at least once a week were considered. Therefore, we generated a new variable (media exposure) by combining the three media sources. Media exposure was labeled ‘Yes’ if respondents had exposure to at least one of the media channels at least once a week and labeled ‘No’ otherwise.

We generated the women empowerment (decision-making) variable from the three decision-making questions. The questions are about the women’s decision-making regarding their health care, large household purchases, and visits to family and relatives. We then classified the number of decision-making the women participated in as none (women who did not participate in any of the decisions), one to two (respondents who mentioned that they took decisions alone or with their husband/partner on one to two of the decision-making variables), and three (respondents who mentioned that they make decisions alone or with their husband/partner on all of the decision-making variables).

### Data processing and management

We performed the data processing and analysis using STATA 15 software. Before any statistical analysis, we weighted the data using sampling weight, primary sampling unit, and strata. Sampling weights are adjustment factors applied to each case in tabulations to adjust for differences in the probability of selection cases in a sample due to either design or chance. In DHS surveys, the sample is selected with unequal probability to expand the number of cases available (hence, reducing sample variability) for certain areas or subgroups for which statistics are needed. In this case, weights need to be applied when tabulations are made of statistics to produce the proper representation. We conducted cross-tabulations and summary statistics to describe the study population.

### Statistical analysis

Because the DHS data is hierarchical, women within a cluster may be more similar to each other than women in the other cluster. As a result, a violation of the premise of independent observations and equal variance across clusters may happen. To obtain a credible standard error and unbiased estimate, a sophisticated statistical model that accounts for between-cluster variability is necessary.

Multilevel logistic regression models allow us to take into account the clustered nature of the data, investigate sources of variations within and between clusters, describe which variables predict individual differences, and describe which variables predict cluster-level differences [23, 24]. We used a multilevel mixed-effect logistic regression to account for the dichotomous nature of the outcome variable. We compared the Models using the Akaike and Bayesian Information Criteria (AIC and BIC), and we chose a mixed effect model with the lowest Information Criteria (AIC and BIC).

In the bi-variable multilevel mixed-effect logistic regression model, we independently analyzed individual and community-level variables associated with modern contraceptives. For the final individual and community-level model adjustments, we incorporated variables that were statistically significant at a p-value of 0.25 in the bi-variable multilevel mixed-effect logistic regression analysis. In the multivariable multilevel mixed-effect analysis, we considered variables with a p-value of less than 0.05 as significant factors associated with modern contraceptive use. We used the intra-class correlation coefficient (ICC) to determine whether a multilevel model was appropriate and how much of the total variation in the response can be explained by clustering [24].

Four different models were used in this study analysis. Model 1 was the null model, which did not include any exposure variables and was used to validate community variance and give evidence to assess random effects at the community level. The second model was a multivariable model that was adjusted for individual-level variables, while the third model was modified for community-level characteristics. Model 4 included potential candidate factors from both the individual and community levels [24, 25]. Finally, we utilized fixed effects (a measure of association) to estimate the relationship between modern contraceptive use and independent variables and reported an odds ratio with a 95% confidence range. We utilized community-level variance with standard deviation and intra-cluster correlation coefficient (ICC) as measures of variation (random effects).

## Results

### Socio-demographic characteristics

There were a total of 34,292 couples in the nine East African countries. However, we included a total of 32,925 respondents in the final analysis after excluding respondents or partners who were sterilized (750) and declared infecund (617). The highest number of the study participants were from Ethiopia 6564 (19.4%), and the smallest were from Tanzania 1536 (4.5%). The majority (14375, 42.4%) of the women lie in the age group of 25–35 years. Most (26524, 78.3%) of the study participants were rural residents, while 16076 (47.5%) had a primary level of education (Table 1).

**Table 1:** Weighted socio-demographic characteristics of married women in East Africa n = 32,925 (weighted total = 33,880).

Characteristics	Weighted frequency (%)	Modern contraceptive use	
		Yes (%)	No (%)
**Modern contraceptive use (%)**		15477 (45.7)	18402 (54.3)
**Age **			
Less than 25 years	8671(25.6)	3690(42.6)	4980(57.4)
25-35 years	14375(42.4)	7151(49.7)	7224(50.3)
More than 35 years	10833(32.0)	4635(42.8)	6198(57.2)
**Residence**			
Urban	7355(21.7)	3936(53.5)	3418(46.5)
Rural	26525(78.3)	11540(43.5)	14984(56.5)
**Country**			
Burundi	3717(10.9)	868(23.4)	2849(76.6)
Ethiopia	6564(19.4)	2482(37.8)	4082(62.2)
Madagascar	4463(13.2)	2010(45.1)	2452(54.9)
Malawi	3442(10.2)	2001(58.1)	1441(41.9)
Rwanda	2931(8.7)	1751(59.8)	1179(40.2)
Tanzania	1537(4.5)	483(31.4)	1054(68.6)
Uganda	2323(6.9)	830(35.7)	1493(64.3)
Zambia	5415(16.0)	2666(49.2)	2749(50.8)
Zimbabwe	3488(10.3)	2385(68.4)	1103(31.6)
**Education level**			
No education	8508(25.1)	2767(32.5)	5741(67.5)
Primary	16076(47.5)	7598(47.3)	8478(52.7)
Secondary	8069(23.8)	4455(55.2)	3613(44.8)
More than secondary	1227(3.6)	657(53.6)	570(46.4)
**Husband Education level**			
No education	6271(18.5)	2095(33.4)	4176(66.6)
Primary	16211(47.9)	7222(44.6)	8989(55.4)
Secondary	9132(26.9)	4962(54.3)	4169(45.7)
More than secondary	2266(6.7)	1198(52.9)	1068(47.1)
**Religion**			
Protestant	10323(30.5)	4783(46.3)	5540(53.7)
Catholic	9460(27.9)	4125(43.6)	5335(56.4)
Jova witness	4333(12.8)	2411(55.7)	1922(44.3)
Adventist	3500(10.3)	1319(37.7)	2181(62.3)
Muslim	2944(8.7)	1501(51.0)	1443(49.0)
Other	3320(9.8)	1338(40.3)	1983(59.7)
**Wealth status**			
Poor	13527(39.9)	5420(40.1)	8107(59.9)
Middle	6864(20.3)	3274(47.7)	3590(52.3)
Rich	13489(39.8)	6783(50.3)	6706(49.7)
**Occupation**			
Occupied	23848(70.4)	11005(46.2)	12843(53.8)
Unoccupied	10032(29.6)	4472(44.6)	5559(55.4)
**Sex of household head**			
Male	32395(95.6)	14756(45.6)	17639(54.4)
Female	1485(4.4)	722(48.6)	764(51.4)
**Media exposure**			
Yes	14695(43.4)	7590(51.6)	7104(48.4)
No	19185(56.6)	7887(41.1)	11298(58.9)

### Medical and reproductive characteristics

Nineteen percent of the women reported that health workers visited them within 12 months before the study period. The majority (61.9%) of the women in this study were empowered to make decisions regarding their health care, large household purchases, and visits to family and relatives. On the other hand, 40% of the women mentioned they had a big problem regarding distance to health facilities. Nearly one-third (30.4%) of women had given birth to more than five children (Table 2).

**Table 2:** Weighted reproductive characteristics of married women in East Africa, n = n = 32,925 (weighted n = 33,880).

Characteristics	Total frequency (%)	Modern contraceptive use		
		Yes (%)		No (%)
**Visited by health worker in last 12 months**				
Yes	6577(19.4)	3290(50.0)		3287(50.0)
No	27303(80.6)	12186(44.6)		15115(55.4)
**Visited health facility in last 12 months**				
Yes	22368(66.0)	10,523(47.0)		11844(53.0)
No	11512(34.0)	4954(43.0)		6558(57.0)
**Women’s Empowerment (Participation in Decision Making)**				
**Final say on making large household goods’ purchases**				
Yes	24499(72.3)	11566(47.2)		12,932(52.8)
No	9381(27.7)	3910(41.7)		5470(58.3)
**Final say on visits to family or relatives**				
Yes	27332(80.7)	12825(46.9)		14506(53.1)
No	6548(19.3)	2651(40.5)		3896(59.5)
**Final say on woman’s health care**				
Yes	26573(78.4)	12456(46.9)		14116(53.1)
No	7307(21.6)	3021(41.3)		4286(58.7)
**Number of decisions that women participate in**				
None	3315(9.8)	1220(36.8)		2095(63.2)
One to two of them	9578(28.3)	4366(45.6)		5213(54.4)
Three of the decisions	20987(61.9)	9892(47.1)		11095(52.9)
**Total children ever born (gravidity)**				
0	2001(5.9)	224(11.2)		1777(88.8)
1-4	21581(63.7)	11139(51.6)		10442(48.4)
5+	10298(30.4)	4114(40.0)		6184(60.0)
**How soon couples want more children**				
Wants within two years	5537(16.3)	1621(29.3)		3916(70.7)
Wants after two years	13179(38.9)	6723(51.0)		6457(49.0)
Does not want	13020(38.4)	6299(48.4)		6721(51.6)
Undecided	2144(6.4)	835(39.0)		1309(61.0)
**Couple’s desire for children (weighted n = 33,871)**				
Both want same	16178(47.8)	7909(48.9)		8270(51.1)
Husband wants more	7458(22.0)	3194(42.8)		4264(57.2)
Husband wants fewer	3778(11.2)	1890(50.0)		1888(50.0)
Do not know	6457(19.0)	2485(38.5)		3973(61.5)
**Births in the last five year**				
No birth	8933(26.4)	3200(35.8)		5733(64.2)
1 birth	15356(45.3)	8418(54.8)		6939(45.2)
2+births	9591(28.3)	3860(40.3)		5731(59.7)
**Recent delivery with C-section** (weighted n = **24,908)**				
Yes	1263.6774 (5.1)	747(59.2)		515(40.8)
No	23644 (94.9)	11513(48.7)		12131(51.3)
**Distance to health facilities**				
Big problem	13621(40.2)	5666(41.6)		7955(58.4)
Not big problem	20259(59.8)	9811(48.4)	10447(51.6)	
**Ever had terminated pregnancy**				
Yes	5196(15.3)	1990(38.3)		3205(61.7)
No	28684(84.7)	13486(47.0)		15197(53.0)

### Factors associated with modern contraceptive use

In multi-variable multilevel logistic regression analysis, the odds of modern contraceptive use among women in the age group 25–35 years were higher by 20% (AOR = 1.20, 95% CI: 1.12, 1.29) compared to women aged less than 25 years. The odds of modern contraceptive use among women in primary, secondary, and more than secondary levels of education were higher by 36% (AOR = 1.36, 95%CI: 1.26, 1.47), 31% (AOR = 1.31, 95%CI: 1.19, 1.46) and 36% (AOR = 1.36, 95%CI: 1.13, 1.64), respectively compared to women with no education. On the other hand, the odds of modern contraceptive use were 14% higher (AOR = 1.14, 95%CI: 1.03, 1.26) when the husband’s education is secondary level compared to no education.

The odds of modern contraceptive use among women with no big problem regarding the distance to health facilities were higher by 14% (AOR = 1.14, 95%CI: 1.08, 1.21) compared to their counterparts. The odds of modern contraceptive use among women who participate in one to two and three of the decision-making variables were higher by 15% (AOR = 1.15, 95% CI: 1.04, 1.27) and 16% (AOR = 1.16, 95%CI: 1.06, 1.28), respectively compared to women who do not participate in any of the decisions. The odds of modern contraceptive use among women with media exposure were 12% higher (AOR = 1.12, 95% CI:1.05, 1.19) than their counterparts. The odds of modern contraceptive use among women with middle and rich wealth status were 31% (AOR = 1.31, 95% CI:1.21, 1.40) and 25% (AOR = 1.25, 95% CI: 1.15, 1.35) higher compared to women with poor wealth status, respectively. The odds of modern contraceptive use among occupied women were higher by 24% (AOR = 1.24, 95% CI: 1.16, 1.32) compared to their counterparts. Compared to women with protestant religion, the odds of modern contraceptive use among women with Catholic and Muslim religion were higher by 10% (AOR = 1.10, 95% CI:1.01, 1.19) and 26% (AOR = 1.26, 95%CI: 1.11, 1.42) respectively, while Adventists were lower by 22% (AOR = 0.78, 95% CI: 0.68, 0.89). The odds of modern contraceptive use among women with one to four and more than five children were 6.56 (AOR = 6.56, 95% CI:5.53, 7.79) and 6.18 (AOR = 6.18, 95% CI: 5.10, 7.48) times higher respectively, compared to women with no children. On the contrary, the odds of modern contraceptive use were 27% less among women with a history of termination of pregnancy (AOR = 0.73, 95% CI: 0.68, 0.78) compared to their counterparts. The odds of modern contraceptive use among women living in Malawi, Rwanda, Zambia, and Zimbabwe were 1.86 (AOR = 1.86, 95% CI: 1.59, 2.18), 1.70 (AOR = 1.70, 95% CI:1.45, 2.01), 1.24 (AOR = 1.24, 95% CI: 1.06, 1.45), and 3.43 (AOR = 3.43, 95% CI: 2.87, 4.09) times higher as compared to women living in Ethiopia, respectively. Conversely, the odds of modern contraceptive use among women living in Burundi, Tanzania, and Uganda were lower by 69% (AOR = 0.31, 95% CI: 0.27, 0.37), 36% (AOR = 0.64, 95% CI: 0.51, 0.79) and 37% (AOR = 0.63, 95% CI: 0.54, 0.75) compared to women living in Ethiopia, respectively. Furthermore, rural residents were 12% less likely (AOR = 0.88, 95% CI: 0.80, 0.97) to use modern contraceptives compared to urban residents (Table 3).

**Table 3:** Multi-variable multilevel logistic regression analysis of factors associated with modern contraceptive utilization in East Africa.

Variables	Models				
	Model 1 AOR(95%CI)	Model 2 AOR(95%CI)	Model 3 AOR(95%CI)		Model 4 AOR(95%CI)
**Age of respondent**					
Less than 25 years	**-**	1.00	**-**		1.00
25-35 years	**-**	1.25(1.17, 1.34)**	**-**		1.20 (1.12, 1.29)**
More than 35 years	**-**	1.01(0.92, 1.12)	**-**		0.94 (0.85, 1.03)
**Highest Education Level**	** **		** **		** **
No education	**-**	1.00	**-**		1.00
Primary	**-**	1.59(1.47, 1.71)**	**-**		1.36 (1.26, 1.47)**
Secondary	**-**	1.73(1.57, 1.91)**	**-**		1.31(1.19, 1.46)**
More than secondary	**-**	1.86(1.55, 2.24)**	**-**		1.36(1.13, 1.64)**
**Husband education level**	** **		** **		** **
No education	**-**	1.00	**-**		1.00
Primary	**-**	1.22(1.13, 1.32)**	**-**		1.08(0.99, 1.17)
Secondary	**-**	1.44(1.30, 1.59)**	**-**		1.14(1.03, 1.26)**
More than secondary	**-**	1.30(1.11, 1.51)**	**-**		1.05(0.90, 1.22)
**Media exposure**					
No	**-**	1.00	**-**		1.00
Yes	**-**	1.15(1.08, 1.22)**	**-**		1.12(1.05, 1.19)**
**Wealth status**	** **	** **	** **		** **
Poor	**-**	1.00	**-**		1.00
Middle	**-**	1.25(1.16, 1.34)**	**-**		1.31(1.21, 1.40)**
Rich	**-**	1.15(1.07, 1.24)**	**-**		1.25(1.15, 1.35)**
**Occupation**					
Unccupied	**-**	1.00	**-**		1.00
Occupied	**-**	1.08(1.01, 1.15)**	**-**		1.24(1.16, 1.32)**
**Religion**	** **	** **	** **		** **
Protestant	**-**	1.00	**-**		1.00
Catholic	**-**	1.00(0.92, 1.07)	**-**		1.10(1.01, 1.19)**
Jova witness	**-**	1.49(1.35, 1.65)**	**-**		0.94(0.84, 1.05)
Adventist	**-**	1.05(0.93, 1.18)	**-**		0.78(0.68, 0.89)**
Muslim	**-**	1.45(1.29, 1.63)**	**-**		1.26(1.11, 1.42)**
Other	**-**	0.88(0.79, 0.98)**	**-**		0.91(0.80, 1.04)
**Total number of children**					
0	**-**	1.00	**-**		1.00
1-4	**-**	6.86(5.77, 8.15)**	**-**		6.56(5.53, 7.79)**
5+		6.07(5.01, 7.35)**			6.18(5.10, 7.48)**
**Distance to health facilities**					
Big problem	**-**	1.00	**-**		1.00
Not big problem	**-**	1.13(1.07, 1.20)**	**-**		1.14(1.08, 1.21)**
**Ever had terminated pregnancy**					
No	**-**	1.00	**-**		1.00
Yes	**-**	0.71(0.66, 0.77)**	**-**		0.73(0.68, 0.78)**
**Visited by a health worker in the last 12 months**					
No	**-**	1.00	**-**		1.00
Yes	**-**	1.12(1.05, 1.20)**	**-**		1.05(0.98, 1.13)
**Visited health facility last 12 months**					
No	**-**	1.00	**-**		1.00
Yes	**-**	0.95(0.89, 1.00)	**-**		1.02(0.96, 1.08)
**Couple’s desire for children**					
Both want same	**-**	1.00	**-**		1.00
Husband wants more	**-**	0.82(0.77, 0.88)**	**-**		0.83(0.77, 0.88)**
Husband wants fewer		1.01(0.93, 1.10)			1.00(0.92,1.09)
Do not know	**-**	0.75(0.69, 0.80)**	**-**		0.76(0.70, 0.81)**
**The number of decisions that women participate in**					
None	**-**	1.00	**-**		1.00
One to two of them	**-**	1.22(1.11, 1.35)**	**-**		1.15(1.04, 1.27)**
Three of the decisions		1.24(1.13, 1.36)**			1.16(1.06, 1.28)**
**Births in the last five year**					
No birth		1.00			1.00
1 birth		1.64(1.52, 1.76)**			1.68(1.56, 1.81)**
2+births		1.07(0.98, 1.16)			1.16(1.06, 1.26)**
**How soon couples want more children**					
Wants within two years		1.00			1.00
Wants after two years		2.43(2.24, 2.64)**			2.42(2.23, 2.63)**
Does not want		2.34(2.14, 2.55)**			2.19(2.00, 2.39)**
Undecided		1.42(1.25, 1.61)**			1.37(1.21, 1.55)**
**Country**					
Ethiopia	**-**	-	1.00		1.00
Burundi	**-**	-	0.47(0.40, 0.54)**		0.31(0.27, 0.37)**
Madagascar			1.36(1.20, 1.56)**		1.05(0.90, 1.22)
Malawi	**-**	-	2.60(2.27, 2.98)**		1.86(1.59, 2.18)**
Rwanda	**-**	-	2.85(2.47, 3.29)**		1.70(1.45, 2.01)**
Tanzania	**-**	-	0.75(0.63, 0.89)**		0.64(0.51, 0.79)**
Uganda	**-**	-	0.93(0.80, 1.08)		0.63(0.54, 0.75)**
Zambia	**-**	-	1.63(1.43, 1.86)**		1.24(1.06, 1.45)**
Zimbabwe			4.02(3.46, 4.67)**		3.43(2.87, 4.09)**
**Place of residence**	** **				** **
Urban	**-**	-	1.00		1.00
Rural	**-**	-	0.74(0.69, 0.81)**		0.88(0.80, 0.97)**
**Random effects**					
Community variance	0.92(0.04)	0.71 (0.03)	0.55 (0.02)	0.51 (0.02)	
ICC%	21.78%	17.67%	14.27%	13.46%	
**Model comparison**					
AIC	44072.39	40822.39	42819.57	**39905.37**	
BIC	44089.19	41116.45	42911.99	**40275.04**	

Note: ** = significant at P-value < 0.05; ICC = Intra-class Correlation Coefficient; AOR = Adjusted Odds Ratio; AIC = Akaike Information Criteria; BIC = Bayesian Information Criteria

## Discussion

This study assessed modern contraceptive use and its determinants among married women in East Africa. The overall prevalence of modern contraceptives was 45.68%, 95% CI (45.2, 46.2). Woman’s age (25-35 years), maternal education, husband education, media exposure, wealth status, occupation, religion, the total number of children ever born, distance to health facilities, history of termination of pregnancy, couple’s desire for children, number of decisions that women participate in, births in the last five years, living country and place of residence were significantly associated with modern contraceptive use in Eastern Africa.

The overall prevalence of modern contraceptive use in the current study (45.68%) was higher than a previous study conducted in the Eastern Africa region (35.99%) [26] and a systematic review in Sub-Saharan Africa (SSA) [27]. The reasons for the variation could be differences in sample size, study populations, and the study periods. On the other hand, the prevalence of modern contraceptive use in this study was lower than a previous study reported from Southern SSA(57.0%) [28] and Malawi (75%) [27]. The possible reason for the difference might be variations in study settings and the availability of the contraceptive. The present finding shows that the highest modern contraceptive use was in Zimbabwe (68.4%), similar to the previous study in the Eastern Africa region [26].

In comparison to Ethiopia, the odds of modern contraceptive use were 69%, 36%, and 27% lower in Burundi, Tanzania, and Uganda, respectively. The reason could be a high number of married women in Burundi and Tanzania and a high number of aged women in Uganda.

Modern contraceptive use was lower in rural residents compared with their urban counterparts in the present study. The reason could be a lack of accessibility to the services and the unavailability of contraceptives for women in rural areas. Additionally, women in rural areas might lack awareness, which prevents them from using the contraceptive method they choose.

Similar to previous studies [20, 29, 30], modern contraceptive use was higher among occupied women compared to unoccupied ones. The reason might be the inadequacy of time, which hinders occupied women from deciding to have children.

In the current study, modern contraceptive use was 17% lower if the husband wanted more children compared to couples who had the same desire for children. This finding contradicts a study from the 2019 Liberia Demographic and Health Survey [31]. Besides, modern contraceptive use was 2.19 times higher among couples who do not want more children compared to couples who want more children within two years. This finding was in line with a study conducted in Senegal [32]. This finding indicates that the less desire for children, the more tendency to use contraceptives.

The probability of modern contraceptive use was 27% lower among women with a history of termination of pregnancy in comparison to their encounters, which is not similar to earlier research reports from Liberia [31]. The reason might be a psychological fear of the association of contraceptives with the termination of pregnancy.

The likelihood of modern contraceptive use was 1.14 times higher among women who do not suffer from distance to health facilities, which contradicts a study from sub-Saharan Africa [26]. The reason could be being near the health facility improves access to contraceptives.

Based on the present study result, the use of modern contraceptives was significantly higher among followers of the Muslim religion compared to Protestants. This finding contradicts studies conducted in 29 sub-Saharan African countries, where Muslim religion followers had a lower tendency to use modern contraceptives than Christians [33]. The reason for the discrepancy could be due to the difference in the stratification of the variable and the reference variable used. In our study, we analyzed the association of modern contraceptive use with religion by classifying Christianity into subcategories, while it was broadly analyzed by classifying religion into Muslim and Christian in the later study.

In the current study, the odds of modern contraceptive use were higher among women with the highest educational level. This report is in line with a multilevel analysis done in sub-Saharan Africa (SSA) countries from 2006 to 2018 [26, 34] and a systematic review and meta-analysis conducted on modern contraceptive use in SSA [35]. The rationale might be that educated women would read newspapers, watch the news, and use various social media platforms to learn about the advantages of modern contraception. Furthermore, educated couples may exhibit positive health-seeking behavior and utilize health services, including family planning options. Besides, the use of modern contraceptives among married women whose partners had completed secondary education was 1.14 times higher than those whose partners had no education. This finding is similar to the previous studies that showed that a higher level of education of the husband encourages women’s contraceptive usage [36, 37].

In this study, women’s empowerment in decision-making significantly influences the odds of modern contraceptive use, which is in line with the previous studies [26, 38]. An increase in women’s decision-making skills and support from their partners increase their willingness to use modern contraceptives [39].

Modern contraceptive use was significantly higher among women with a higher wealth index compared to their counterparts. This finding is consistent with a study conducted in 36 sub-SaharanAfrican countries using the DHS data [26]. Apossible reason might be better financial resources can lead to better access to reproductive health care, including the use of contemporary contraceptives [40].

In this study, the odds of modern contraceptive use were 1.12 times higher among women who have media exposure compared to women who have no media exposure. This finding is in line with a systematic review and meta-analysis conducted on 31 sub-Saharan African countries between 2005 and 2015 [41] and a multilevel analysis done on 36 sub-Saharan African countries between 2006 and 2018 [26]. The possible reason might be mass media exposure can increase the accessibility of information relating to reproductive health care information, including modern contraceptive use. The media is also essential in raising awareness to resolve social and cultural barriers that may limit the use of modern contraceptives [42].

In the present study, modern contraceptive use was significantly higher among women with a higher number of children compared to women with no children. This finding is consistent with several prior studies [14, 43, 44] indicating that women with more children intend to space their pregnancies.

### Strengths and limitations of the study

We analyzed data from nationally representative multi-country surveys, and the use of a high sample size for the analysis is a strength of this study. However, the study’s findings do not prove a causal link between the outcome variable and independent variables because the design used was cross-sectional. Furthermore, because the study is a secondary data analysis, additional predictors, including the standard of treatment (counseling about side effects and supply accessibility) and cultural factors, were not included in the analysis. We advise conducting a longitudinal study using primary data to acquire information on all pertinent aspects to address the limitations of the cross-sectional design and the absence of several crucial variables in this study.

## Conclusion

The use of modern contraceptives among married women in East Africa was low, while the prevalence varies based on nation and residence. The prevalence contraceptive usage was highest in Zimbabwe and lowest in Burundi, while it was lower in rural areas than urban areas. This study identified several factors that are significantly associated with modern contraceptive use. Accordingly, women’s age, maternal education level, husband’s education level, media exposure, wealth status, occupation, religion, the total number of children ever born, distance to health facilities, history of termination of pregnancy, couple’s desire for children, women’s participation in decision making, living country and place of residence were significantly associated with modern contraceptive use. The findings of this study indicate that numerous preventable factors lower the use of modern contraceptives. As a result, we recommend interventions such as encouraging formal education, enhancing access to health care, raising awareness about the use of modern contraceptives, improving women’s empowerment in decision-making, and raising media exposure for better use of modern contraceptives. Furthermore, we recommend taking the experience of countries with better modern contraceptive use for proper policy action and program interventions.

## Supporting information

S1 File (PDF)
